# Placebo Analgesia, Acupuncture and Sham Surgery

**DOI:** 10.1093/ecam/neq030

**Published:** 2011-03-13

**Authors:** Tao Liu, Cui-ping Yu

**Affiliations:** ^1^Department of Traditional Chinese Medicine, 2nd Teaching Hospital, Norman Bethune Medical School, University of Jilin, 218 Ziqiang Street, Changchun 130041, Jilin Province, China; ^2^Department of Anatomy, Changchun Medical College, Changchun, Jilin Province, China

## Abstract

Invasive procedures, such as surgery and acupuncture, are likely better than the others in terms of eliciting placebo analgesia. Understanding how invasive procedures can elicit enhanced placebo responses may provide new insights into mechanisms underlying placebo analgesia. In this essay, it is argued that sensory, cognitive and emotional factors are major determinants of the magnitude of placebo analgesia. Sham surgery and acupuncture are good examples of placebo interventions, which generate robust placebo responses through simultaneously manipulating such three factors.

## 1. Introduction

Although the intensity of pain correlates well with the degree of noxious stimulation, the perception of pain is not a linear phenomenon, reflecting the signal from peripheral nociceptors [1, 2], but rather heavily shaped by the psychosocial context in which pain presents itself [3–5]. A striking example is placebo analgesia which has increasingly been a research focus [6–10] for the potential of leading to the development of novel approaches to the control of chronic pain [11, 12]. Placebo analgesia does not happen all the time and varies greatly across clinical and experimental contexts. The essential question has been how and when placebo analgesia is powerful and if placebo analgesia can have clinical relevancy. Interestingly, sophisticated, invasive and painful interventions such as surgery and acupuncture are thought to have enhanced placebo effects [13–16]. Understanding how invasive procedures can elicit enhanced placebo responses may provide new insights into mechanisms underlying placebo analgesia.

Acupuncture is an ancient Chinese therapy with its mode of action unclear and efficacy inconclusive [17, 18]. Although acupuncture has been defined as simplistically as the practice of inserting one or more needles into specific sites on the body surface for therapeutic purposes, converging evidence from theory basis, clinical practice and clinical and basic science research of acupuncture supports that acupuncture has more than needling with its underlying cognitive and emotional context being an integral part of this therapy [19]. But individual contributions of different components of acupuncture to its therapeutic effects remain to be identified.

In the following, it is argued that cognitive, sensory and emotional factors codetermine the magnitude of placebo analgesia. Hence, enhanced placebo analgesia can be elicited through manipulating such three factors. Sham surgery and acupuncture are good examples of placebo interventions, which generate robust placebo responses through simultaneously engaging sensory, cognitive and emotional factors.

## 2. Factors That Determine the Magnitude of Placebo Analgesia

There is no doubt that changes in pain cognition (e.g. expectations and beliefs for pain) are fundamental factors underlying placebo analgesia. Expectations for reduced pain trigger placebo analgesia, and higher levels of less negative expectations tend to produce larger placebo analgesic effects [20]. However, expectations alone have not always been found to produce placebo effects [21], and the magnitude of placebo analgesia has been reported to be subject to the influences of other factors. For example, an early study showed larger placebo effects with more intense pain, indicating that sensory factors contribute significantly to the magnitude of placebo analgesia [22]. Moreover, it has been reported that individuals are more likely to demonstrate a placebo effect when they are feeling anxious than when they are not [23, 24], supporting that emotional factors such as anxiety are other major determinants of the magnitude of placebo analgesia [25]. Why have there been these different approaches to mechanisms underlying placebo analgesia? One possible explanation is that, although it is a critical factor underlying placebo analgesia, expectation is not likely to operate alone [26, 27], but sensory and emotional factors are also involved in expectation-based placebo analgesia. In fact, the rationale to assume that sensory, cognitive and emotional factors are involved in placebo analgesia lies in the consideration that placebo analgesia is the consequence of the same painful stimulus presented in a changed cognitive and emotional context. Placebo analgesia is triggered by cognitive factors but heavily shaped by concurrent sensory and emotional factors—that is, sensory, cognitive and emotional factors are major determinants of the magnitude of placebo analgesia. These three factors vary greatly across clinical and research contexts, which may account for the considerable variability in the placebo effect itself.

## 3. Factors That Contribute to Placebo-Induced Activation of Endogenous Opioids

### 3.1. Role of Endogenous Opioids in Placebo Analgesia

Although both opioid and non-opioid mechanisms are responsible for placebo analgesia [28], an emerging idea is that expectation-based placebo effects are opioid-mediated, but conditioned placebo effects may depend on other mechanisms [7, 29–31]. Early pharmacological studies show that placebo analgesia is antagonized by the opioid antagonist naloxone [32, 33]. Additionally, the opioid-mediated analgesic placebo response is enhanced with proglumide, a cholecystokinin antagonist that modulates opioid activity [34]. Neuroimaging studies reveal that placebo affects endogenous opioid activity in a number of *μ*-opioid receptor regions that play central roles in pain and affect [7, 35], and the same regions of the brain are affected by both a placebo and the opioid agonist remifentanil, indicating a shared brain mechanism involved in placebo- and opioid-induced analgesia [9]. These findings support a strong role for endogenous opioids in placebo analgesia, and the extent to which endogenous opioids are activated may determine the magnitude of placebo analgesia. However, little is known about how opioid mechanisms come into play in expectation-based placebo analgesia, and factors that determine the functional state of placebo-activated endogenous opioids remain to be identified.

### 3.2. Individual Contributions of Sensory, Cognitive, and Emotional Factors to Placebo-Induced Activation of Endogenous Opioids

Given that, as outlined above, cognitive, sensory and emotional factors codetermine the magnitude of placebo analgesia, it appears reasonable to propose that these three factors are major determinants of the magnitude of placebo-induced activation of endogenous opioids. When and only when these three factors are simultaneously brought into full play, can endogenous opioids be most effectively activated.

One of the more informative discoveries about placebo-activated endogenous opioids is that highly spatial-specific placebo responses, which are totally blocked by the opioid antagonist naloxone, can only be obtained in the site of the ongoing pain which is the target of spatially directed expectation of pain reduction [20, 29, 36]. This suggests that placebo-activated endogenous opioids have a precise and somatotopic organization and, more importantly, the location of the ongoing pain determines how endogenous opioids are somatotopically activated, hence in which body parts placebo analgesic effects occur.

Taken together, sensory, cognitive and emotional factors contribute differently to placebo analgesia through determining different aspects of the functional state of placebo-activated endogenous opioids: the intensity of painful stimulation, the level of expectations for pain reduction and the degree of fear and anxiety induced by pain codetermine of the magnitude of placebo-induced activation of endogenous opioids; in addition, the location of the ongoing pain determines how endogenous opioids are somatotopically activated, hence the location of placebo analgesic effects.

### 3.3. How Can Robust Placebo Analgesia Be Induced in Specific Body Parts?

This identification of individual contributions of sensory, cognitive and emotional factors to placebo-induced activation of endogenous opioids actually reveals how robust placebo analgesia can be induced in specific body parts through manipulating these three factors. A painful stimulus applied in an enhanced cognitive (positive expectations for this painful stimulus) and emotional (fear and anxiety induced by the application of this painful stimulus) context could, as outlined above, to a greater extent activate somatotopically organized endogenous opioids, thus producing enhanced placebo analgesia in the location of the stimulus. More importantly, if such a cognitively and emotionally engaged painful stimulus is applied to the site of a clinical pain, then robust placebo analgesia can be produced in the site of this clinical pain—that is, the experienced intensity of this clinical pain can be greatly reduced. Reasoning in this way, a given clinical pain can be treated by applying another pain to specific body parts in an enhanced cognitive and emotional context. For example, knee pain can be treated by the application of another pain as a therapy to the knee. But is there a clinically used placebo therapy that depends on effectively engaging endogenous opioids through simultaneously manipulating sensory, cognitive and emotional factors? Acupuncture is a likely candidate.

## 4. Is There a Clinically Used Placebo Therapy?

Acupuncture is an ancient Chinese therapy, most commonly used for pain control, involving inserting needles into specific parts of the body. However, its mechanisms of action are not well understood, and controversy regarding its clinical efficacy remains [37]. Initially, research attention has therefore been solely focused on the needle insertion due to the beliefs that if acupuncture is efficacious it must be specifically due to this technical procedure. The essential question concerning the efficacy of acupuncture is therefore that if acupuncture generates specific effects superior to its placebo control. Randomized placebo-controlled trials are therefore introduced to assess the specific efficacy of needle insertion on the basis of the biomedical consideration that efficacious therapy is therapy that proves to be superior to its placebo control in a randomized controlled trial. When the majority of randomized controlled trials of acupuncture fails to show effects beyond a placebo response [38, 39], acupuncture has been dismissed as nothing more than placebo [40]. However, to say that acupuncture is not better than placebo does not mean that it does nothing. In fact, the essential issue concerning the effectiveness of acupuncture is not if acupuncture has more than placebo effects, but rather how acupuncture can produce such robust placebo effects as to have clinical relevancy.

### 4.1. Is Acupuncture an Enhanced Placebo?

Research data support the critical role of beliefs and expectations in the effectiveness of acupuncture. A recent study concluded that the effectiveness of acupuncture was significantly associated with higher outcome expectations [41], and therefore how patients are informed about the trial of acupuncture they take part in may greatly influence trial outcomes [42]. Another study went even a step further reporting that patient's beliefs and expectations (determined by the perceived assignment to real acupuncture or a placebo) had a greater impact on treatment outcomes than did the actual treatment itself [43]. Furthermore, research findings from neuroimaging studies on acupuncture analgesia show that acupuncture may act on similar neural networks as placebo (expectations for reduced pain) in producing pain relief [44–47]. Indeed, it has been suggested that it is not meaningful to split acupuncture into specific and non-specific elements and the use of placebo and sham controlled trial designs in evaluating acupuncture may generate false-negative results [48]. In line with this suggestion, considering the critical role played by expectations and beliefs in the effectiveness of acupuncture, it has been proposed that acupuncture is actually an enhanced placebo and necessarily comprises three integral and indispensable components, which are of the same importance in ensuring the effectiveness of acupuncture, including needle insertion (spatial-specific painful stimulation), culture-enhanced positive beliefs and benefit expectations for needle stimulation (cognitive context), and fear and anxiety induced by the perceived invasiveness of needle insertion (emotional context) [19]. This new view of acupuncture as an enhanced placebo warrants a new look at specific and non-specific elements involved in the clinical administration of acupuncture and challenges the present consideration of randomized controlled trials as gold standard in evaluating the efficacy of acupuncture, for it is unlikely meaningful to evaluate the effectiveness of a placebo therapy such as acupuncture with placebo-controlled trial designs.

### 4.2. Cognitive and Emotional Context Underlying Acupuncture

Culture can have profound impacts on medicine through shaping individuals' beliefs [49]. Acupuncture is definitely a culture-based therapy embedded in unique beliefs and rituals [37, 50, 51], allowing patients to have elevated positive beliefs and benefit expectations on this therapy. Furthermore, comprehensive and intimate acupuncturist–patient relationship is another factor contributing to enhanced benefit expectations for this therapy [52]. Furthermore, fear and anxiety comprise the characteristic emotional context in which acupuncture is administered. Acupuncture is, at any rate, an invasive procedure which can never fail to cause considerable levels of fear and anxiety in the patient while receiving this treatment, even for those who repeatedly receive this treatment. Actually, this invasiveness induced fear and anxiety can make great impacts on the patient's mind: a wide range of adverse effects occurring before/during needle administration can be attributable to the patient's fear and anxiety, including, just list a few, severe nausea, actual fainting, severe dizziness, heavy sweating and vomiting [53].

### 4.3. Why Does Acupuncture Induce Enhanced Placebo Analgesia?

These observations strongly point to the assumption that acupuncture depends on the administration of invasive procedures (i.e., needle insertion that produces spatial-specific painful stimulation) in an enhanced cognitive and emotional context to make therapeutic effects. Such three integral components of acupuncture, as outlined above, can most effectively activate somatotopically organized endogenous opioids and, thus, generate robust placebo effects in specific parts of the body (the location of the target pain). This is consistent with studies showing that acupuncture analgesia is largely mediated by endogenous opioids [54–56]. Most importantly, according to traditional Chinese medicine (TCM) meridian theory, spatial-specific needle stimulation induces therapeutic effects only in specific parts of the body, not only in the site of needle stimulation (local effects) but also at some distance from the site of stimulation along a specific meridian (distal effects). That is, pain of different locations and tissues origins needs to be treated by inserting needles into different parts of the body. Acupuncture is therefore a good example of clinically used placebo therapies which produce clinically relevant placebo responses through simultaneously manipulating sensory, cognitive and emotional factors. That explains why acupuncture is provided in such a sophisticated manner and how clinical provision of acupuncture can elicit robust placebo effects.

**Figure 1:**
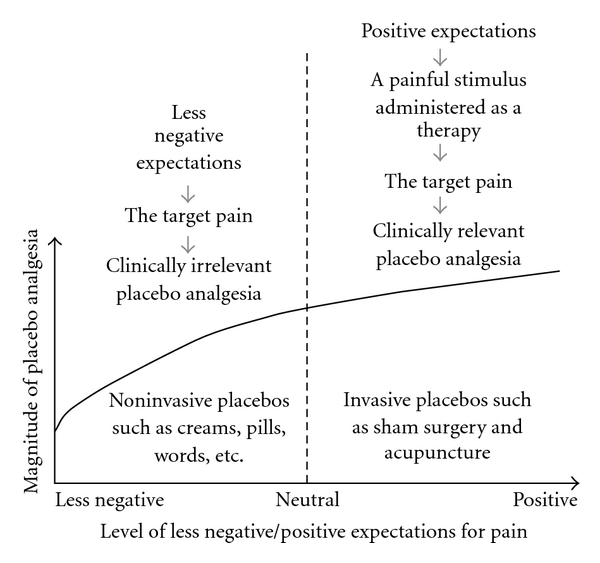
Compared with non-invasive placebos (such as placebo creams, pills and words), which induce less negative (neutral at best) expectations for the target pain, invasive placebos such as sham surgery and acupuncture induce positive (neutral at least) expectations for a painful stimulus administered as a therapy, thus producing clinically relevant placebo analgesia.

The level of placebo expectations, which is a major determinant factor of the magnitude of placebo analgesia, varies greatly across clinical and experimental contexts. At one end of the scale are less negative expectations for the target pain (i.e., expectations for the reduction of the target pain) as with non-invasive placebos such as placebo cream, pills, words and practitioner-patient relationships, whereas at the other end of the scale are positive expectations (i.e., positive beliefs and benefit expectations) for a painful stimulus administered as a therapy as with invasive placebos such as acupuncture. There is no doubt that positive expectations are definitely different in nature from, and obviously of a higher level than, less negative expectations. Compared with that less negative expectations for the target pain do not elicit clinically relevant placebo analgesia, positive expectations for a spatial-specific painful stimulation induced by invasive procedures could more effectively activate somatotopically organized endogenous opioids, thus generating enhanced placebo analgesia of clinically relevancy in the location of the target pain (Figure 1). In short, enhanced placebo responses of acupuncture do not result directly from less negative expectations for the target pain but rather indirectly from positive expectations for needle stimulation administered as a therapy. Although pain is undoubtedly an aversive and negative experience and can hardly be associated with positive expectations, clinical administration of acupuncture successfully induces positive beliefs and benefit expectations for a therapeutic pain through which endogenous opioids can be most effectively activated. Acupuncture can therefore be viewed as depending on “positive” pain to cure negative pain.

## 5. Why Does Sham Surgery Generate Robust Placebo Analgesia?

This new account of enhanced placebo analgesia of acupuncture is supported by further evidence from randomized sham-controlled trials of invasive interventions for pain. Clinical trials of surgery have seldom included placebo surgery as a control, owing to ethical concerns [57, 58]. However, occasionally used sham-controlled surgical trials dramatically showed that sham surgical procedures could be as effective as real procedures in terms of the improvements in subjective measures such as pain and function [59–62]. These studies demonstrate how much of the success of operative procedures for pain reported by observational trials is due to the placebo effect [63].

But why can sham surgery elicit especially robust placebo analgesia of clinical relevancy? A possible explanation is that sham surgery depends on the administration of invasive procedures such as skin incisions and sutures (which produce spatial-specific painful stimulation) in an enhanced cognitive and emotional context to generate robust placebo analgesia.

### 5.1. Cognitive and Emotional Context Underlying Sham Surgery

Surgery is particularly meaningful in a culture rich in machines and tools and therefore induces a profound meaning response in modern medical practice [49, 64, 65]. What features clinical administration of surgery is enhanced positive beliefs and benefit expectations for invasive procedures: surgeons are among the elite of medical practitioners; surgical procedures usually have compelling rational explanations, which drug treatments often do not; in addition, the invasive nature itself of surgery is also an important contributing factor to enhanced positive beliefs and benefit expectations. Furthermore, there is no doubt that fear and anxiety comprise the characteristic emotional context in which surgery is administered, for surgery is obviously invasive and can cause inevitable pain and most likely blood shedding.

### 5.2. Why Does Sham Surgery Elicit Enhanced Placebo Analgesia?

On the basis of these considerations, it is reasonable to propose that sensory (spatial-specific painful stimulation induced by sham procedures), cognitive (positive beliefs and benefit expectations for the painful stimulation) and emotional (fear and anxiety induced by the perceived invasiveness of surgical procedures) factors are simultaneously engaged by the administration of sham surgery. A combination of these three factors, as outlined above, could most effectively activate somatotopically organized endogenous opioids to produce clinically relevant analgesic effects in specific body parts (the location of the target pain). In this regard, interestingly, sham surgery and acupuncture can be considered to be analogous in terms of a spatial-specific painful stimulus administered in an enhanced cognitive and emotional context. This proposition is convincingly supported by research findings from recent sham-controlled trials of invasive interventions for pain of various locations. A large-scale, randomized controlled trial of arthroscopic surgery for knee osteoarthritis reported that sham operations (which produce spatial-specific painful stimulation) administered in the skin of the knee, which elicit robust analgesic effects in the knee, are no less effective for knee pain than real procedures [60]. Not coincidently, a recent high-quality randomized controlled trial of vertebroplasty for painful osteoporotic vertebral fractures drew similar conclusions that sham operations administrated in the affected vertebral body can as effectively reduce pain in that area as real procedures [62].

## 6. Conclusion

Previous studies focused on the role of cognitive factors (expectations and beliefs) in placebo responding. However, the present hypothesis argues that sensory, cognitive and emotional factors are simultaneously at work during placebo analgesia. Furthermore, clinically relevant placebo analgesia can be produced in specific body parts through the manipulation of such three factors as in the case of acupuncture and sham surgery. That explains why invasive procedures, such as acupuncture and sham surgery, are better than the others in terms of eliciting placebo analgesia. Acupuncture is a clinically used placebo therapy, theory basis and clinical administration of which highlight one of the best, if not the best, manners through which enhanced placebo effects can be produced in specific body parts (the site of the target pain). Research into the mode of action of acupuncture will shed direct light on neural mechanisms underlying placebo analgesia and vice versa.
